# AI-enabled evaluation of genome-wide association relevance and polygenic risk score prediction in Alzheimer's disease

**DOI:** 10.1016/j.isci.2024.109209

**Published:** 2024-02-12

**Authors:** Daniel E. Platt, Aldo Guzmán-Sáenz, Aritra Bose, Subrata Saha, Filippo Utro, Laxmi Parida

**Affiliations:** 1 IBM T. J. Watson Research Center, Yorktown Heights, New York, NY, USA; 2 Pfizer, Pearl River, New York, NY, USA

**Keywords:** Human Genetics, Quantitative genetics, Biocomputational method, Association analysis, Machine learning

## Abstract

GWAS focuses on significance loosing false positives; machine learning probes sub-significant features relying on predictivity. Yet, these are far from orthogonal. We sought to explore how these inform each other in sub-genome-wide significant situations to define relevance for predictive features. We introduce the SVM-based RubricOE that selects heavily cross-validated feature sets, and LDpred2 PRS as a strong contrast to SVM, to explore significance and predictivity. Our Alzheimer’s test case notoriously lacks strong genetic signals except for few very strong phenotype-SNP associations, which suits the problem we are exploring. We found that the most significant SNPs among ML and PRS-selected SNPs captured most of the predictivity, while weaker associations tend also to contribute weakly to predictivity. SNPs with weak associations tend not to contribute to predictivity, but deletion of these features does not injure it. Significance provides a ranking that helps identify weakly predictive features.

## Highlights

• Probe sub-genome-wide significant SNPs using significance with predictivity
• We introduce an SVM pipeline with heritability-based feature ranking
• We find significance tends to mark more predictive features

## Graphical abstract

**Figure:**
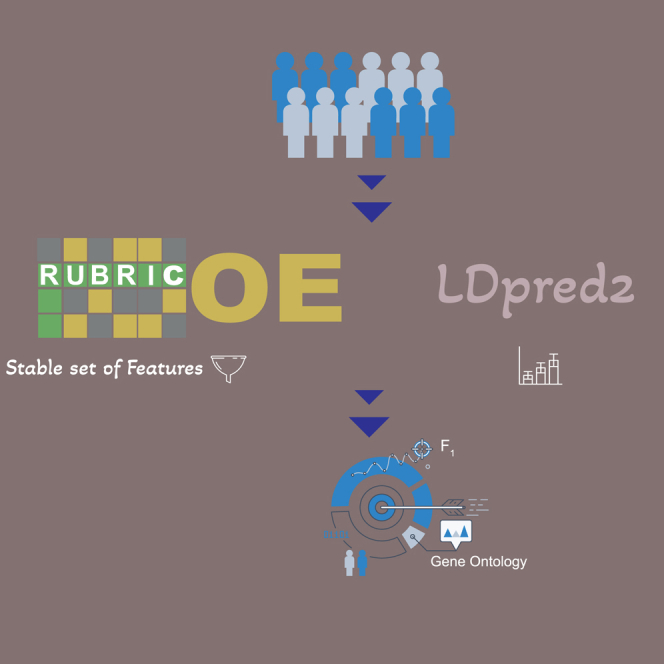

## Introduction

In the search for genetic causes of disease, the assembly of a cohort represents a random sampling of the population. Within that random sample, individuals with various alleles and phenotypes will be collected with variations in proportions due to the stochastic variation in the sampling process. One question is whether overrepresentation of an allele state among those with a phenotype vs. those without is really meaningful, or is an artifact of sampling. It is possible to estimate the probability that such an overrepresentation could have emerged by chance instead of being due to an actual association (which is the null hypothesis). Such a probability is called its significance. Thresholds are set to exclude false negatives (significance thresholds), but at the cost of excluding a number of true active alleles, resulting in false negative rejections. One basic problem in standard genome-wide association studies (GWAS) is that thresholds required to guarantee no neutral SNPs were accepted above a significance threshold across the entire genome. For new whole-genome sequence (WGS) technologies, those thresholds are very severe, resulting in large numbers of false negatives.

There is hope that machine learning (ML) and polygenic risk score methods (PRS) may discover variants that may account for the missing heritability of some diseases. These methods offer the possibility of probing false negative SNPs below genome-wide significance thresholds, at the expense of including false positives, by seeking allele sets that maximize predictive power for a phenotype given a set of alleles (referring to measures of recall and precision,^1^ including measures such as $F_{1}$, confusion matrices, false positive rates, etc.). However, the predictivity and statistical significance of simple linear regression coefficients are tightly tied to each other, which we show in the ranking section. However, given large numbers of features, even relatively strongly significant SNPs may be lost among false positives. A primary goal of this study is to understand the relationship between the statistical significance of features, and their predictivity in ML and PRS approaches. The question is relevant in that therapeutics require identification of targetable pathways likely active in the population in order to be useful.

A major premise of GWAS and other similar approaches is that they implicate genes and pathways leading to the etiology of disease and therapeutic targets. A challenging aspect of Alzheimer’s disease (AD) is that genome-wide significant SNPs do not account for the heritable variation in risk,^2^^,^^3^ with some efforts seeking some of that missing variability in epistatic coexpressions.^4^ An attractive idea is to capture relevant SNPs excluded by severe genome-wide thresholds by probing for SNPs below those genome-wide thresholds that maximize predictivity. PRS analyses share much in common with ML approaches in adopting this approach.

Unfortunately, false positives among non-genome-wide significant SNPs spuriously predict disease variation appearing as overfitting in PRS and ML models that include large numbers of variants.^5^^,^^6^ ML investigations, seeking the most reliably predictive variables, employ methods such as regularization and cross-validation to weed out SNPs with less generalizable predictive information. Typical ML approaches employ cross-validation with feature ranking and selection performed on a training dataset, and scoring of those selected models on a test dataset. The approach is finding interest in GWAS-like studies as well.^7^^,^^8^

The nonlinearity of artificial intelligence approaches obscures the contribution of individual SNPs to predictivity.^9^ At the same time, these approaches seek to solve similar problems, such as overfitting. In this study, we seek to understand how model selection for ML and PRS pipelines relates to GWAS-based significance and effect strength (odds ratios) estimations, what is different about the approaches’ selections, and how their predictive power varies. We note that some formulations of PRS start by ranking SNPs by significance, and select thresholds according to some measure of predictivity, possibly including heritability. The methods are very similar to support vector machine (SVM). We note LDpred^10^ and LDpred2^11^ take a different approach, computing scores according to assigned weights.

To that end, we contrast predictive power vs. significance, in LDpred2 and in a SVM solution we call RubricOE (as an exemplar of SVM/ML approaches for predictive omics epidemiology). The contrast between approaches offers parallel but not identical rankable metrics for comparison. We use the heritability/significance ranking from ridge regression we used in our pipeline, RubricOE, and we ordered the LDpred2 weights as a comparable ranking. Both of these are predictivity-related scales.

These approaches to feature selection cover more diversity in methodology, while being extendable in computational scale. Curiously, while ML literature focuses on feature importance in prediction, there is little connection to feature significance.

AD, with its notoriously short list of strongly associated SNPs, serves as a test case that can be used to probe associations between various models and their contribution to predictivity precisely because of AD’s short list.

To this extent, it is important to recognize that this study is not a standard comparison between approaches (e.g., LDpred2 and RubricOE); instead, the focus is on understanding how significance and effect relate to the predictivity in each of the two methodologies—RubricOE and LDpred2—which were selected to span approaches that have been applied to solve similar problems in distinct ways. So the primary comparisons for both methods are against GWAS significance instead of a direct comparison between methods. Having said this, we do explore how RubricOE and LDpred2’s selection yields different features in their response to significance, and we compare them on predictivity vs. significance accordingly.

### RubricOE

**Figure 1:**
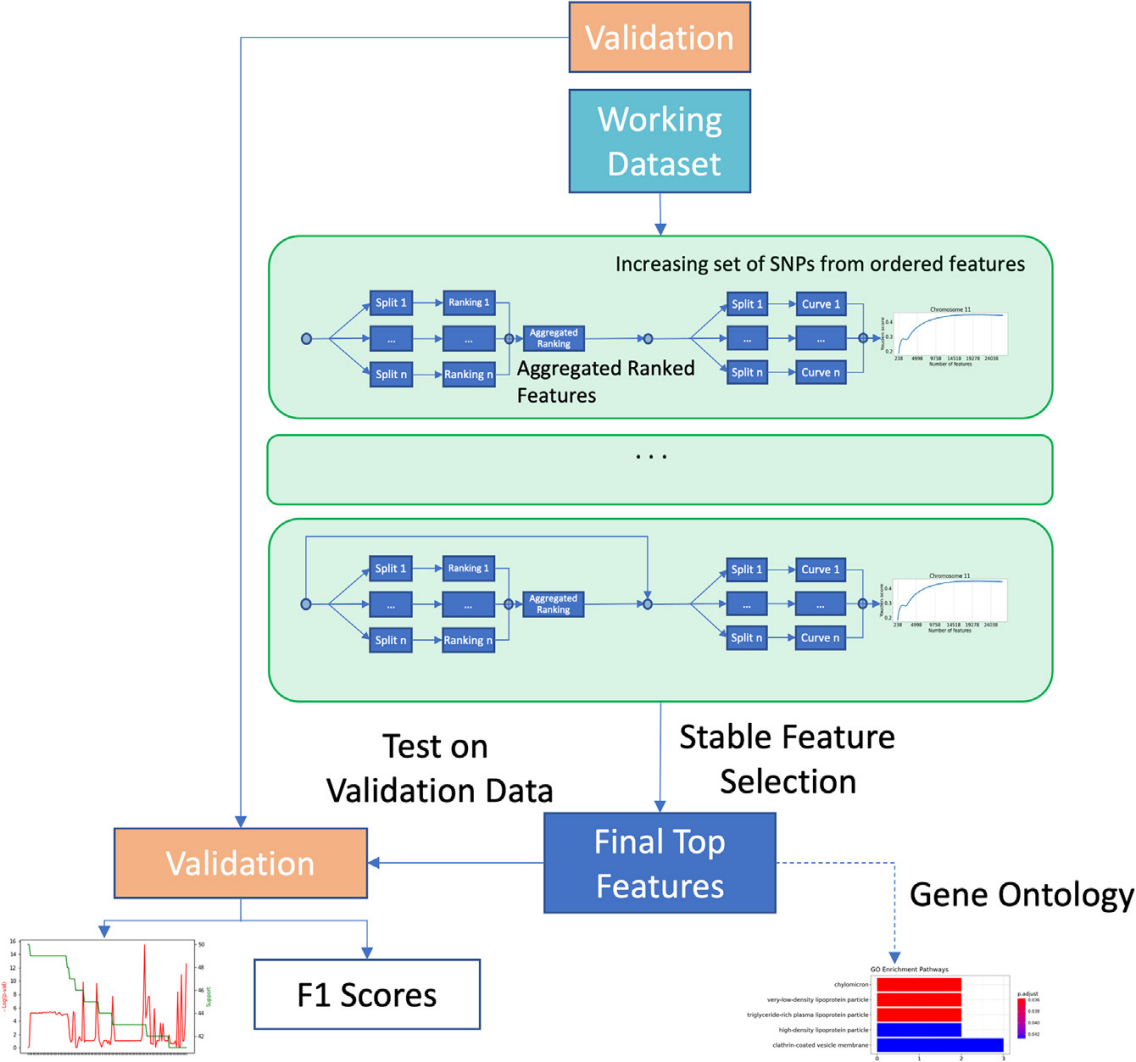
Overview of RubricOE pipeline We begin by splitting the entire dataset into working and validation datasets, then performing feature ranking and scoring multiple times across multiple splits and aggregate results, and finally we validate. The bottom right arrow indicates that the output of the pipeline could be used for Gene Ontology computations.

RubricOE is a cross-validated SVM pipeline with feature ranking described in the ranking section, and multiple levels of cross-validation, with an overview shown in Figure 1. The primary methodological adaptation is ranking features by heritability within the context of a Ridge regularized regression.

First, we set aside validation data, which does not play a part in any subsequent analysis by the pipeline.

From the working dataset, we generate multiple splits of the data. First, we rank features using heritability estimated from regularized linear regressions. We then score features against test sets by using models comprised of leading ranked SNPs with linear kernel SVMs to yield Youden^12^ scores. This second part is performed with multiple data splits as well. Top scoring SNPs are selected according to their Youden scores, and the characteristics of the Youden curve. These steps (after the initial split into validation/working data) are performed several times to select SNPs that appear in multiple iterations. SNP stability is characterized by their absolute frequency counting their appearance in multiple cross-validation replications. Figure 1 also shows a potential use of the outputs of RubricOE for Gene Ontology computations.

Figure S1 shows a schematic view of the pipeline. RubricOE has been implemented in python and it is included in the Geno4SD framework as open source at: https://github.com/ComputationalGenomics/Geno4SD/.

### RubricOE feature ranking

In what follows, we order features by estimating their contribution to heritability^13^ by extending predictability in standard linear regression^14^ to identifying heritability in ridge regression.^15^

Phenotypes or disease outcomes *y* are measured for a row of samples representing individual subjects, each predicted by columns representing characteristics *X* related to disease such as SNPs and available relevant elements such as age, sex, diet, family history, etc. These columns may be scaled genotypes (values {0,1,2}), or binary values representing sex, or age above a threshold, diet, and exercise, or could be scaled “quantitative” traits representing continuous variables, such as BMI, if available. Such a linear model would posit y=Xm+ϵ, where *m* indicates a contribution of the column in *X* to the prediction of *y*, and ϵ represents random variations due to sampling or other sources with E(ϵ)=0, $\text{cov}\left(\epsilon ,\epsilon^{T}\right)=\Delta y^{2}$, a diagonal matrix, and an offset is included as a column $X_{0}=1$.

A measure of goodness of fit for *m* can be constructed as $E^{2}=\epsilon^{T}\Delta y^{-2}\epsilon ={\left(y-Xm\right)}^{T}\Delta y^{-2}\left(y-Xm\right)$. Defining the least-squares coefficient estimator as $\bar{m}={\left(X^{T}\Delta y^{-2}X\right)}^{-1}X^{T}\Delta y^{-2}y\text{,}$ and the residual error as $E_{res}^{2}=y^{T}\Delta y^{-2}y-y^{T}\Delta y^{-2}X{\left(X^{T}\Delta y^{-2}X\right)}^{-1}X^{T}\Delta y^{-2}y\text{,}$ then $E^{2}=E_{res}^{2}+{\left(m-\bar{m}\right)}^{T}\left(X^{T}\Delta y^{-2}X\right)\left(m-\bar{m}\right)$. So the minimum error is $E_{res}^{2}$, obtained when $m=\bar{m}$. The covariance is $\text{cov}\left(\bar{m},{\bar{m}}^{T}\right)={\left(X^{T}\Delta y^{-2}X\right)}^{-1}$.

The residual may be written: $E_{res}^{2}=y^{T}\Delta y^{-2}y-{\bar{m}}^{T}{\left[\text{cov}\left(\bar{m},{\bar{m}}^{T}\right)\right]}^{-1}\bar{m}=y^{T}\Delta y^{-2}y-{\left(X\bar{m}\right)}^{T}\Delta y^{-2}\left(X\bar{m}\right)$. So the predictive power of the individual features is effectively tied to their *Z* scores, which establishes a strong connection between significance and predictivity. This motivated the current study. The amount of predictive power, feature by feature, is characterized as the fraction of variation $y^{T}\Delta y^{(}-2)y$ accounted for by ${\left(X\bar{m}\right)}^{T}\Delta y^{(}-2)\left(X\bar{m}\right)$. Since the expression includes the impact of covariance between features, highly correlated features cannot be treated independently in computing their predictivity to *y*. These effects may be managed by using linkage disequilibrium (LD) pruning or clumping.

In underdetermined systems, $X^{T}\Delta y^{-2}X$ is likely to have null eigenvalues and will not be invertible. Contributions to $E^{2}$ by *m* in the directions of those null eigenvectors will be zero, so the regression offers no information about variations in *m* and the covariance diverges in those directions. Further, since the non-null eigenvectors completely span the sampled space, the value of $E_{res}^{2}$ is 0, and the set of features splines any set of values to be predicted: a situation called overfitting. One approach to control for this overfitting is by noting that splined $\bar{m}$ tends to have larger values. The impact of larger values is controlled by applying an $L_{2}$ regularization term to the regression so that of $E^{2}={\left(y-Xm\right)}^{T}\Delta y^{-2}\left(y-Xm\right)+Cm^{T}m$. We chose $L_{2}$ over $L_{1}$ due to its tractability for error propagation. The first impact of the regularization term is to ensure that there are no divergent components of $\text{cov}\left(\bar{m},{\bar{m}}^{T}\right)$, and second to define a measure of “unusual” for “unusually large” components of *m* typical of splining. In this case, $\bar{m}={\left(X^{T}\Delta y^{-2}X+CI\right)}^{-1}X^{T}\Delta y^{-2}y$, $E_{res}^{2}=y^{T}\Delta y^{-2}y-{\left(X\bar{m}\right)}^{T}\Delta y^{2}\left(X\bar{m}\right)-C{\bar{m}}^{T}\bar{m}$, $E^{2}=E_{res}^{2}+{\left(m-\bar{m}\right)}^{T}\left(X^{T}\Delta y^{-2}X+CI\right)\left(m-\bar{m}\right)$, and $\text{cov}\left(\bar{m},{\bar{m}}^{T}\right)={\left(X^{T}\Delta y^{-2}X+CI\right)}^{-1}X^{T}\Delta y^{-2}X{\left(X^{T}\Delta y^{-2}X+CI\right)}^{-1}$. Note that the residual error contains another contributor $C{\bar{m}}^{T}\bar{m}$ beyond the predictive ${\left(X\bar{m}\right)}^{T}\Delta y^{-2}\left(X\bar{m}\right)$ reflecting the impact of the regularization term. Further ${\left(X\bar{m}\right)}^{T}\Delta y^{-2}\left(X\bar{m}\right)\neq {\bar{m}}^{T}{\left[\text{cov}\left(\bar{m},{\bar{m}}^{T}\right)\right]}^{-1}\bar{m}$, so the predictive power is less tightly connected to the *Z* scores of the features. We use the individual diagonal components from ${\left(X\bar{m}\right)}^{T}\Delta y^{-2}\left(X\bar{m}\right)$ for LD pruned or clumped sets to estimate the heritability of individual SNPs in the context of a ridge regularized linear regression.

Estimators for the coefficients we use for ranking are:

$$\bar{m}={\left(X^{T}\Delta y^{-2}X+CI\right)}^{-1}X^{T}\Delta y^{-2}y\text{,}$$

$$\text{cov}\left(\bar{m},{\bar{m}}^{T}\right)={\left(X^{T}\Delta y^{-2}X+CI\right)}^{-1}X^{T}\Delta y^{-2}X{\left(X^{T}\Delta y^{-2}X+CI\right)}^{-1}\text{.}$$

The residual error yields a quantity tied to the heritability term in simple linear regression, so we select the measures reducing the residual error by each coefficient to rank the SNPs. We use the individual diagonal components from ${\left(X\bar{m}\right)}^{T}\Delta y^{-2}\left(X\bar{m}\right)$ for LD pruned or clumped sets to estimate the heritability of individual SNPs in the context of a ridge regularized linear regression.

The residual may be written: $E_{res}^{2}=y^{T}\Delta y^{-2}y-{\bar{m}}^{T}{\left[\text{cov}\left(\bar{m},{\bar{m}}^{T}\right)\right]}^{-1}\bar{m}=y^{T}\Delta y^{-2}y-{\left(X\bar{m}\right)}^{T}\Delta y^{-2}\left(X\bar{m}\right)$. So the predictive power of the individual features is effectively tied to their *Z* scores, which establishes a strong connection between significance and predictivity. This motivated the current study. The amount of predictive power, feature by feature, is characterized as the fraction of variation $y^{T}\Delta y^{(}-2)y$ accounted for by ${\left(X\bar{m}\right)}^{T}\Delta y^{-2}\left(X\bar{m}\right)$. Since the expression includes the impact of covariance between features, highly correlated features cannot be treated independently in computing their predictivity to *y*. These effects may be managed by using LD pruning or clumping.

Youden’s *J* statistic is defined as the J=sensitivity−falsepositiverate=sensitivity+specificity−1. A plot of *J* scores for ranked data as a function of a ranking threshold in a sensitivity vs. false positive rate will indicate a point where threshold adjustment to include more samples to acquire more true positives starts including more false positives. We proposed to rank features (SNPs) according to the estimate of simple heritability accounted for in a regularized linear regression.

### Cross-validation

For a fraction *f* of biologically active SNPs, a process identifying biologically active SNPs, i.e., an odds ratio threshold, has a probability α of falsely identifying a true SNP, and a probability β of rejecting a biologically active SNP. So, the fraction of SNPs that the test would identify as positive would be f⋅(1−β)+(1−f)⋅α, and the fraction of SNPs identified as being valid is $\frac{f\cdot \left(1-\beta \right)}{f\cdot \left(1-\beta \right)+\left(1-f\right)\cdot \alpha }$. For *N* replications, the proportion of false positives supported in all replications would be $\alpha^{N}$, and for true positives ${\left(1-\beta \right)}^{N}$. So, the fraction of SNPs we would expect to be true positives across *N* replications would behave as $\approx \frac{f\cdot {\left(1-\beta \right)}^{N}}{f\cdot {\left(1-\beta \right)}^{N}+\left(1-f\right)\cdot \alpha^{N}}\to 1$ for *N* large. The chances that replication will be primarily populated with true positives are when $\left(1-f\right)\cdot \alpha^{N}\ll f\cdot {\left(1-\beta \right)}^{N}$. The fraction *f* of biologically active SNPs is likely very small compared to 1 for Alzheimer’s. So, the number of required replications required is where $\left(1-f\right)\cdot \alpha^{N}\ll f\cdot {\left(1-\beta \right)}^{N}$.

Cross-validation is applied heavily in our “RubricOE” pipeline.

### Analysis of RubricOE output

RubricOE feature selection assigns Youden scores to the ranked feature sets. In the final output, RubricOE presents a set of SNPs, each with an associated number of replications in which the SNP appeared (support). We apply a logistic regression to each SNP predicting the phenotype to evaluate OR’s and p values, and contrasted predictivity of the entire set, of those that were supported across all replications, and of the most significant SNPs, where predictivity was evaluated using linear kernel SVMs from sklearn.^16^ Genes associated with the SNPs were obtained from the appropriate version of the gene location table from https://www.ncbi.nlm.nih.gov/genome for GRCh38.

### Analysis of LDpred2 output

LDpred2^11^ performed quality control (QC) to the imputed set, including PCA and LD filtering, and produced a set of β scores describing the association between the selected 22,506 SNPs and the phenotypes, applied to compute the individual’s phenotype scores. LDPred2 offers predictivity measures of correlation and AUC between the individual’s predicted phenotype scores and their actual phenotypes, as well as $F_{1}$. LDpred2 assigns weights to all SNPs using a Gibbs sampler. LDpred2 explores the space of hyperparameters in terms of sampled grids of hyperparameter values. LDpred2 presents weights, β, for each of the full set of SNPs. The “auto” option scores all the SNPs that passed QC. A histogram of the numbers of SNPs vs. their scores shows a very large spike near neutral β=0. Since β=0 represents effects close to neutrality, and a large number of SNPs are essentially neutral, exclusion of SNPs with $\beta \leq 2\times 10^{-4}$ does not affect risk scores for any given individual. We selected all SNPs from any of the 50 bins that represented less than $2\times 10^{-4}$ of the SNPs.

## Results

### Data

Data were acquired on June 26, 2021 from the Alzheimer’s Disease Sequencing Project repository at National Institute of Aging Alzheimer’s Data Storage Site (https://www.niagads.org/adsp/), comprised of data not excluded for use by commercial entities,^17^ release R3 from 14474 subjects consenting to access by commercial entities, excluding family set data, retaining 78815437 SNPs marked as “PASS” by variant quality score recalibration in the GATK suite. SNPs showing systematic missingness were identified and filtered. Phenotypes available in the dataset include ethnicity, race, age, sex, AD status, whether diagnosed on enrollment, acquired it during the study, BRAAK stage, and the APOE4 genotype (determined separately from the WGS analysis). We retained age, sex, and AD status. Females comprised 63.3%, 6,446 of the samples, with 45.9%, 4,365 AD diagnosed subjects. The average age of the subjects was 75.4 years, with a standard deviation of 8.9 years.

For the dataset, MAF was filtered at 0.05, LD filtered with window size of 50 kb, step size 5, $r^{2}$ threshold of 0.5, and HWE threshold of $1\times 10^{-12}$. Beagle was applied for imputation.^18^^,^^19^

### RubricOE feature selection

**Figure 2:**
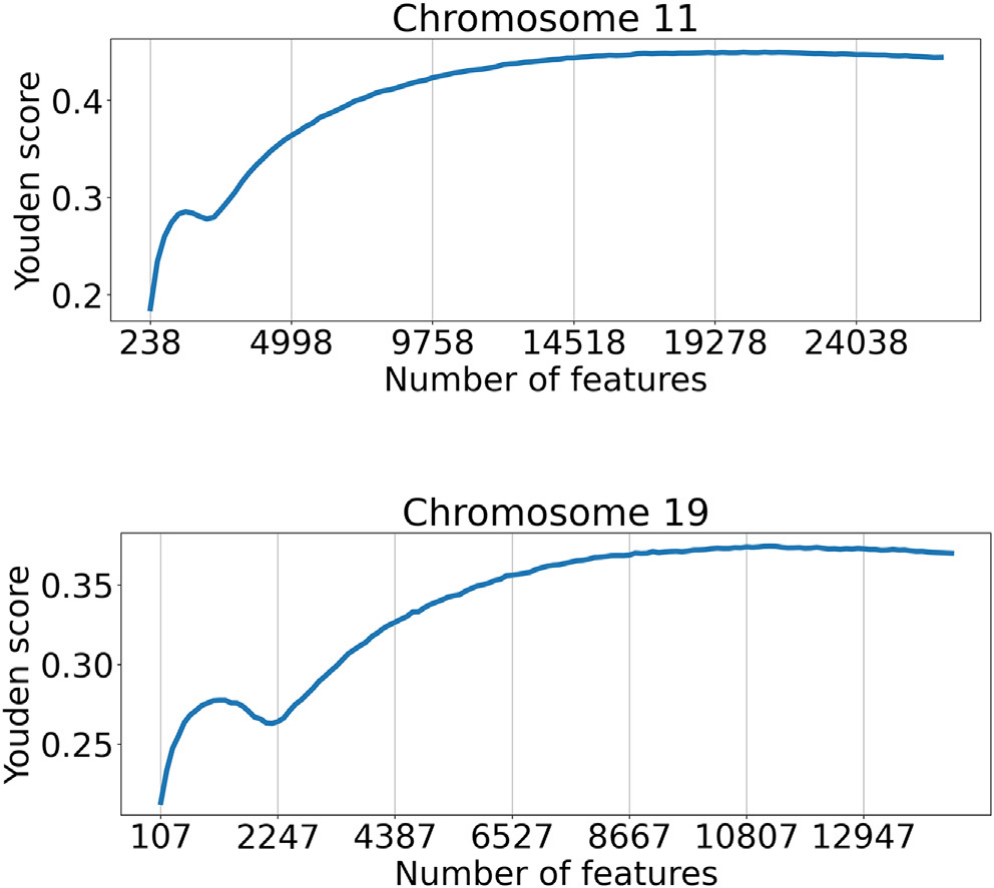
Youden curves for chromosomes 11 and 19 based on heritability ordering from ridge regression

Ranking was based on the estimation of the contribution of each SNP to heritability in a linear ridge regression, as described previously. Ranking-defined nested subsets of features were scored by RubricOE, using a Youden index,^12^ J=sensitivity−(1−specificity), essentially a measure of the amount predicted above what would be expected by chance. In AD, chromosome (chr) 19 contains APOE, whose APOE4 genotype (joint genotype of rs429358 and rs7412) is the most strongly associated with disease expression, with a number of linked and related variants in APOE and nearby genes. Therefore, chr 19 is guaranteed to have a relatively small set of very strong SNP associations. Chr 11 had a number of relatively weak associations that appeared in a meta-analysis with 74,046 samples,^20^ some of which were supported in a much larger meta-analysis with 788,989 samples.^21^ We applied RubricOE to chromosomes (chrs) 11 which has had four variants associated with it in large metastudies,^20^^,^^22^ and to a shuffled set of chr 19 to contrast with chr 11’s weaker SNPs. The Youden indices for chrs 11 and 19 are displayed in Figure 2. The extraction of a Youden curve for a shuffled set was not performed.

Initially, the Youden scores yield a bimodal profile, rapidly accumulating predictivity with included features along the increasing abscissa, up to a local maximum beyond which scores temporarily decrease (the “shoulder”), followed by a larger swell of predictivity scores, as seen in Figure 2. The initial growth for chr 11 is smaller than that for chr 19, suggesting contributions from APOE and linked alleles. Those left shoulders are presumed to show enrichment in true positives compared to the rest of the SNPs, which we take as enriched in true positive candidates for RubricOE input.

### LDpred2 feature selection

**Figure 3:**
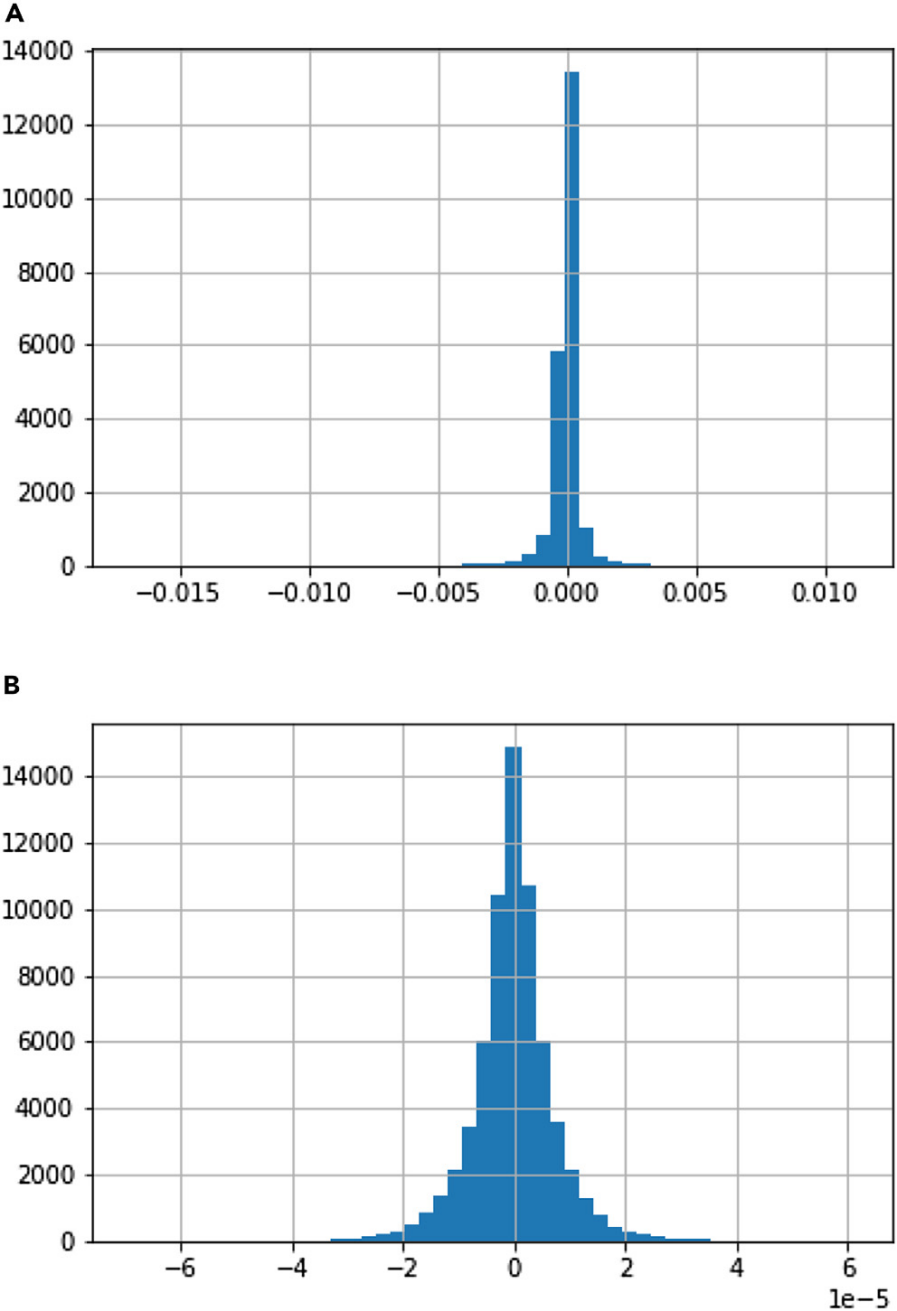
Histogram of the LDpred2 Auto β′s (A) Chr 19 (B) Chr 19 phenotype shuffled.

The histograms (Figure 3) of the LDpred2 Auto scores for the AD data (Figure 3) and shuffled data (Figure 3) show a very striking spike centered around a neutral score β=0 for the unshuffled set, compared to the shuffled set. These weak spike SNPs contribute very little to the predictive value for any given individual. We also note the strength of the signal represented by the β′s is much larger in the unshuffled data compared to the shuffled set. We selected the SNPs outside of the central spike in bins representing less than $2\times 10^{-4}$ of the total number of SNPs, including a number of SNPs that were not genome-wide significant.

### Stable set analysis

We sought to apply a similar analysis for both RubricOE and LDpred2. In both cases, the output is expressed in terms of ranked features. We applied logistic regression to measure the significance of these features, and used SVMs to measure the predictivity of the selected feature sets, with $F_{1}$ being used as the metric for comparison. We tested predictivity of real data vs. shuffled data, and for a strongly associated chromosome (chr 19) and a weakly associated chromosome (chr 11). We also tested predictivity of the whole selected subsets against the significant SNPs within the selected subsets.

**Figure 4:**
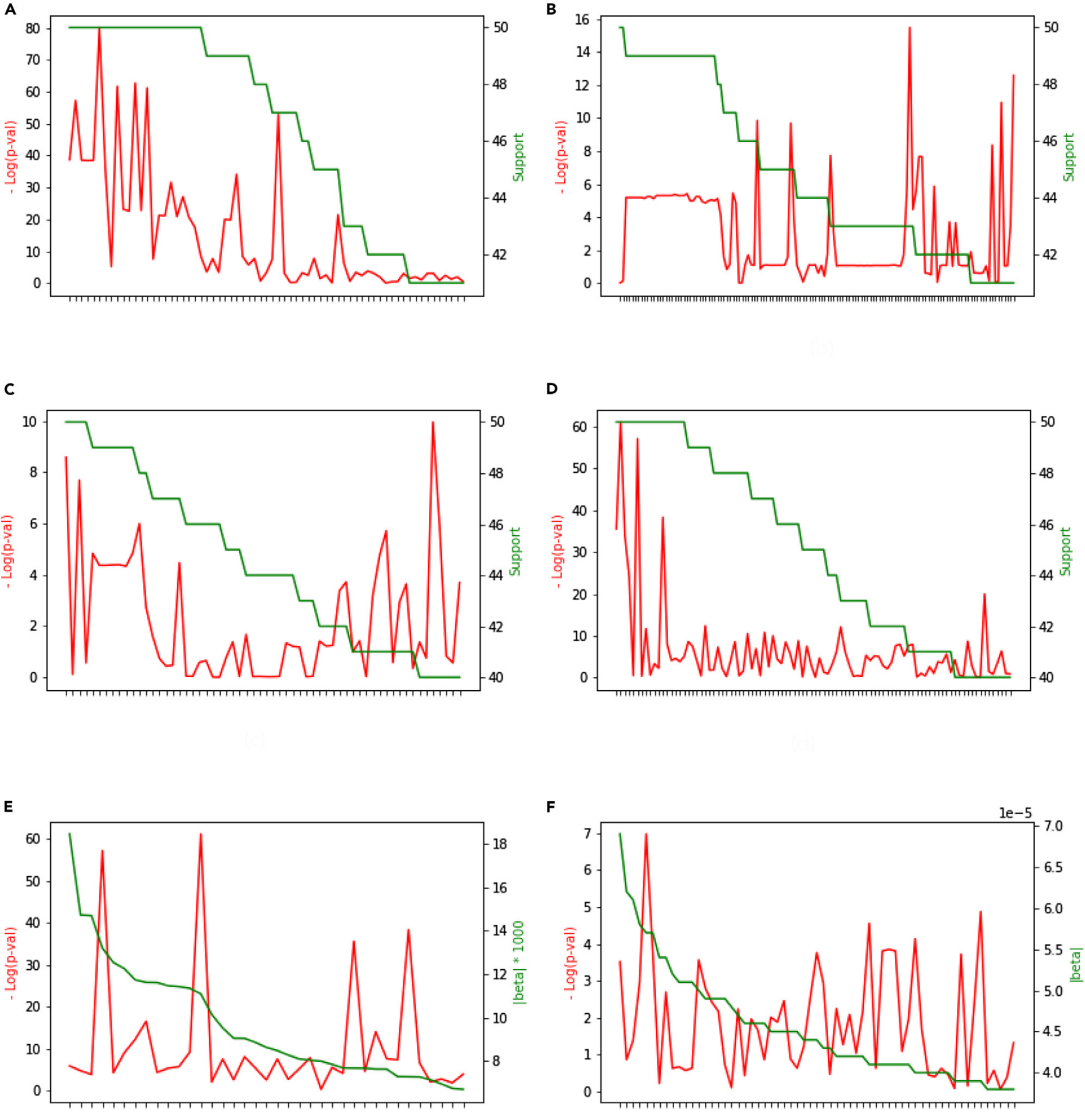
Logistic Regression computed p values and support of selected features ranked by support for chrs 19 and 11, respectively (A) RubricOE Chr 19 with shuffled phenotypes, (B) RubricOE Chr 11, (C) RubricOE on Chr 19 with shuffled phenotypes, (D) RubricOE on Chr 19 with LDpred2 QC applied, (E) LDpred2 Chr 19, and (F) LDpred2 on Chr 19 shuffled. "Support" is the number of RubricOE final cross-validation replications showing the SNP for figures (A–D); we use LDpred2's β as "support" in figures (E and F).

RubricOE assigns to each SNP, selected in the aforementioned feature selection process, the number of stable sets that the SNP was found under cross-validation. The “stable sets” are selected according to the number of replications the features were included, which we call “support.” The features in Figure 4 were ranked by support, displayed in green, and shown in the right-hand scale. The log of the p value computed by logistic regression is displayed in red, according to the scale on the left side. This figure therefor contrasts significance vs. support. Figure 4F serves as a random control by analyzing randomized data. LDpred2’s plot is identical in form, except β replaces support (Figure 4E). The relationship between these SNPs and their p values is displayed in Figure 4.

Chr 19 shows decreasing significance as support declined from a maximum of 50 (Figure 4A) with a rough correlation between them. Specifically, the strength of the log (p values) and density of significant SNPs tend to trend with support, being quenched in low-support SNPs. By contrast, chr 11 has no support reaching a level of 50, and the relationship between significance and support is relatively uncorrelated (Figure 4B). Figure 4D shows RubricOE’s result run on the data preprocessed for LDpred2, showing an expanded range of Figure 4A. Figure 4E shows the results for LDpred2, indicating that inflection points in ordered scores appear to occur at spikes in logistic regression p values where the β scores are strongest. These inflections tend to be lost at weaker β weights. This indicates an interaction between the impact of significance on stronger LDpred2 weights. LDpred2 run on shuffled phenotypes from chr 19 shows much weaker association p values, and much less correspondence between p values and the structure of β (see Tables 1 and 2).

In summary, the relationship between support and significance shown in Figure 4 is generally that more significant and more strongly significant SNPs appear with larger support, or larger β. It is worth noting the shifts in scales between actual chr 19 samples and shuffled or chr 11 samples, as well as the change in dropoff in −log(p) for more weakly supported samples. For example, in Figure 4E, the weaker SNPs are roughly $3\times 10^{6}$ less significant than the SNPs associated with stronger weights β.

Logistic regression results are tabulated together with support for each of the selected SNPs from chr 11 in Table S1 and for chr 19 in Table S4. From the four previously reported SNPs from chr 11, rs3740688, rs7933202, rs3851179, and rs11218343, only two (rs7933202 and rs3851179) passed quality control missingness, and each of those was insignificant in the dataset (p value of 0.1153 and 0.2864, respectively), and was not selected.

The predictive information in the entire selected set was contrasted with subsets with a p value threshold and with a support threshold (Table 2). The p values and supports among the chr 19 selections were much stronger than those of chr 11, so weaker threshold was chosen for chr 11. The predictions are constructed using a linear kernel SVM from sklearn,^16^ with predictivity measured by $F_{1}$ scores.

**Table 1:** |β| histogram bin predictive measures $F_{1}$, OR, and p values

|β| range	$F_{1}$	OR	p value
0.010992–0.012542	0.62	2.489	$1.50\times 10^{-30}$
0.009441–0.010992	0.55	1.314	0.0488
0.007891–0.009441	0.56	1.514	$7.21\times 10^{-6}$
0.006340–0.007891	0.61	2.338	$5.43\times 10^{-26}$
0.004789–0.006340	0.61	2.414	$1.74\times 10^{-29}$
0.003239–0.004789	0.63	2.790	$1.07\times 10^{-39}$
0.001688–0.003239	0.63	2.779	$1.68\times 10^{-40}$

**Table 2:** $F_{1}$ for predictions of AD from RubricOE SNPs

Chromosome	Selected SNPs	Support ≥ 45	−log(p)>30
11	0.58	0.54	0.53
Chromosome	Selected SNPs	Support = 50	−log(p)>50
19	0.61	0.62	0.62
Chromosome	Selected SNPs	Support = 40	−log(p)>5
19 (shuffled)	0.54	0.54	0.53
Chromosome	Selected SNPs	Support = 45	−log(p)>30
19 (PRS QC)	0.65	0.62	0.64
Chromosome	LDpred2 SNPs	|β|≥0.012	−log(p)>30
19 (LDpred2)	0.61	0.61	0.58
Chromosome	LDpred2 SNPs	$\left|\beta \right|\geq 5\times 10^{-5}$	−log(p)>3.5
19 (LDpred2 Shuffled)	0.56	0.55	0.55

In chr 19, the prediction by the most significant SNPs (−log(p)>50) or by the most strongly supported SNPs (support = 50) yielded as much predictivity as the entire set of predictive SNPs ($F_{1}\approx 0.62$). (Note, $F_{1}=2\cdot \frac{precision\cdot recall}{precission+recall}$: the harmonic mean of precision and recall.) This would suggest that the strongest SNPs carried most of the predictivity for the chromosome. In chr 11, none of the SNPs showed either the p value or the support of the strongest SNPs in chr 19. In this case, the strongest p value and most supported SNPs had a predictivity of $F_{1}\approx 0.54$, while the whole collection of selected SNPs had a predictivity of $F_{1}\approx 0.58$, stronger than that of the SNPs with support and stronger p values.

LDpred2 predictivity is broken down by |β| bin in Table 1, displaying $F_{1}$, OR, and p value for each bin range. The ORs are computed on the confusion matrix relating the alleles vs. phenotype. The p values are computed from the standard result that the log of randomly sampled ORs will be normally distributed with mean $\log\frac{n_{DE}n_{\bar{D}\bar{E}}}{n_{D\bar{E}}n_{\bar{D}E}}$, with variance $\sigma_{\log\;OR}^{2}=\frac{1}{n_{DE}}+\frac{1}{n_{\bar{D}\bar{E}}}+\frac{1}{n_{D\bar{E}}}+\frac{1}{n_{\bar{D}E}}$. The bins show a similar behavior as the Youden curves for RubricOE in that the leading edge shows a predictive peak, a dip, and then a swell from the weaker bins. In contrasting significance and support as plotted in Figure 4, we note that greatest evidence for interactions with significance reflected in the inflection points are occurring in regions that contribute relatively less to the overall risk scores. We note this predictivity structure makes it difficult to implicate specific pathways that might be useful for targeting therapies.

## Discussion

AD is notorious for difficulty identifying pathogenic genetic factors promoting the disease in spite of its heritability. The wealth of new information available in next generation sequencing only increases the challenge by raising the threshold required to clearly distinguish true positives from false positives. Approaches to the problem that probe below stiff genome-wide thresholds, seeking to identify falsely rejected SNPs by testing their predictivity, make ML and PRS approaches very attractive. So this paper explores what ML and PRS approaches may have to say about AD and other diseases with similar SNP association profiles.

The answer to that question might best be answered by turning it around: what does the AD SNP association profile reveal about how ML and PRS respond to that kind of signal? In this case, chr 19 is notorious for having a set of strong SNPs generally considered to derive much of their significance from linkage with APOE4. These present a distinctive differentiating character even below genome-wide significance. Meanwhile, chr 11 has relatively weak OR’s,^20^^,^^22^ with little to distinguish weak true SNPs among the false positives from true negatives, and they do not represent predictive power in the selected set. In this sense, they are not distinguishable from overfitting false positives.

A prime question is what relation does predictive SNPs have with possible biological processes in AD or other diseases. In standard linear regression, we show in the Supplement that those SNPs with strongest associations are also those that also account for the most in describing phenotypic variation. To that end, we explored the amount of phenotypic variation accounted by SNPs in ridge regularized regression – heritability, and used those scores in the RubriOE pipeline to rank the SNPs (features) identifying an optimal subset of SNPs to predict phenotypes using Youden indices. Youden scores displayed in Figure 2 reveal a transition from predictivity from stronger SNPs to weaker SNPs, with a separation marked by a notch in the predictivity. It is possible this notch is partly due to the structure of AD associations, which possesses several very strong genotypes, and many weak ones mixed with false positives, with biological effect strength (e.g., odds ratios or regression coefficients) separating the strong vs. weak SNPs. Among weaker SNPs, predictive power of false positives and true positives is essentially indistinguishable. Table 1 shows a corresponding result in LDpred2 β weights, where the strongest weights account for predictivity, which passes through a transition of lowered predictivity below which there is a swell similar to that seen in the RubricOE Youden curves. RubricOE has also been applied to find stable SNPs which led to an increased prediction accuracy of severe COVID-19.^23^

ML and (generic) PRS use cross-validation to exclude false positive SNPs. A short description of the mechanism is presented in the Supplement. RubricOE heavily uses cross-validation to identify stable sets of SNPs. Note that rare alleles will also be lost to cross-validation.

We also provided a null model by shuffling the phenotypes in chr 19 for RubricOE, constructed by shuffling the phenotypes. While spurious associations are expected, RubricOE’s SVM cost functions that discriminated the shuffled phenotype tended to be flat, offering little advantage in minimizing the cost by varying the placement of the SVM margin. Convergence was very slow for the shuffled set. Once identified, they were relatively unlikely to be supported in other cross-validation splits.

A histogram of LDpred2’s β′s histogram (Figure 3) showed much weaker values for β in the shuffled set compared to the unshuffled signal. More, the central spike, marking neutral SNPs, was narrower, but held a higher proportion of SNPs in the real data than in the shuffled set.

Measuring association significance with logistic regression, Figure 4 shows the regression p values against RubricOE’s stability support (number of cross-validation runs that each SNP appeared in the final results) and LDpred2’s β weights. For chr 19, RubricOE’s most strongly supported SNPs associate with the most significant SNPs (Figures 4A and 4D). The appearance of the association in LDpred2 (Figure 4E) shows strong inflection points from stronger to weaker β weights at the significant SNPs. The relationship between association and support or β weights broke down in chr 11 and the shuffled sets (Figures 4B, 4C, and 4F).

We further explored the predictive power of the sets selected by RubricOE, and those with non-negligible weights from LDpred2. This was done by applying a linear kernel SVM to the sets, and scoring with $F_{1}$. For each of the selected sets, we also scored subsets that showed significant regressions, and subsets with the strongest support or |β|. Table 2 shows results of these tests. In RubricOE results, chr 19, whether applied to raw data or imputed data QCed for LDpred2, showed $F_{1}$ scores around 0.6 (noting Figure 4E indicates relatively few significant SNPs were included in the model), whether for the entire set, or for the subset with significant associations identified by logistic regression, or for the subset with the strongest support. Therefore, many of the other SNPs are not contributing significantly more predictive power to the models, but their inclusion does not injure predictivity. They also have persistently survived cross-validation. As such, they represent compatible candidates that may be biologically relevant, but which are not significant in their own right. The RubrcOE and LDpred2 for shuffled phenotypes $F_{1}$’s scored around 0.55 regardless of significance and support. Chr 11 showed a stronger $F_{1}$ for the whole model, but actually lost $F_{1}$ for more strongly supported or more significant SNPs. Chr 19 SNPs that contributed significantly shared by RubricOE and LDpred2 listed in the Supplement include APOE, APOC, and NECTIN2.

RubricOE is relatively expensive to run due to its heavy use of nested cross-validation without offering large improvements to $F_{1}$ over LDpred2. If predictivity was the end of the story, there might be little value to use it. However, it offers a very different model for SNP selection, finding a much more concentrated set of SNPs that yield competitive predictivity. This may be valuable for researchers seeking to implicate relevant pathways for therapeutic targets. It also offers a different dissection of the significance/predictivity from LDPred2, which may help inform researchers of the shape of that space in general in a broader sense.

We conclude that association determined significant SNPs substantially accounts for predictivity of SNP sets selected by ML and PRS approaches. More, the contributions from true positive SNPs with weak associations are both statistically indistinguishable from true negative SNPs, but the tendency for these SNPs to spline, or overfit, predictivity leaves a burden in the end to sort out which of the identified SNPs may be relevant to disease processes. However, the non-significant SNPs that are identified by these approaches tend to have been consistently replicated in RubricOE and LDpred2, and do not injure predictivity by their inclusion. They represent additional candidates that are compatible with the significant SNPs, and may reflect relevant biological processes. Lastly, defining relevance of SNPs in terms of their statistical significance together with their contributions to predictivity helps to understand what ML methods in general (including neural nets), and PRS and support vector machine pipelines specifically may offer to understand AD, as well as to set reasonable expectations for what ML has to say.

### Limitations of the study

The primary purpose of this study is to connect both statistical significance and predictivity and their interaction in the evaluation of relevant features for methods seeking to probe below genome-wide significance. We probed two methods that exemplify ML approaches - SVMs as embodied in our RubricOE pipeline and LDpred2. These are certainly not exhaustive, but do represent some extreme points. Other ML methods do exist, and some of them include both significance and predictive components, such as random forests. The study reported here was intended to be demonstrative more than exhaustive.

AD is challenging since so many of its SNPss associate so weakly with disease,^2^^,^^21^^,^^22^ and heritability has not been fully accounted for. WGS data sampled from more diverse populations offer a chance to contain those features, but identifying them is difficult. The statistical power required for identification of significant association between Alzheimer's disease and SNPs required 700K in imputed metastudies with custom validations. However, the WGS data available provided only around 4K samples with much weaker statistical power, while presenting a vastly expanded number of multiple tests, resulting in much more demanding significance thresholds. This drives the question of what ML methods are probing into features far below genome-wide significance. The methods we selected approach some of those issues in very different ways. Still, pre-analysis validation filters much of the more rare alleles that might be players in inducing Alzheimer’s. Failure to filter, and to impose cross-validation support will result in overfitting which is the result of being unable to distinguish between real biological features, or statistical artifacts virtually guaranteed to be present in these sample sizes. This is a major limitation of this study – yet it was also a primary motive for exploring how these methods performed in probing sub-genome-wide significant data.

## STAR★Methods

### Key resources table

**Table:** 

REAGENT or RESOURCE	SOURCE	IDENTIFIER
**Deposited data**		

Alzheimer’s Dataset	ADSP	Data acquired on June 26, 2021 (release R3) from the https://adsp.niagads.org/

**Software and algorithms**		

RubricOE	This paper	https://github.com/BiomedSciAI/Geno4SD/tree/main/geno4sd/ml_tools/rubricoe
LDpred2	Florian Privé^11^	https://privefl.github.io/bigsnpr/articles/LDpred2.html
sklearn.svm.SVC	Scikit Learn^16^	https://scikit-learn.org

### Resource availability

#### Lead contact

Further information and requests for code should be directed to and will be fulfilled by the Lead Contact Filippo Utro (email: futro@us.ibm.com).

#### Materials availability

This study did not generate new unique reagents.

#### Data and code availability

• Data availability: All data are publicly available to qualified applicants via https://adsp.niagads.org/
• Code: RubricOE (https://doi.org/10.5281/zenodo.10080732) is available at https://github.com/BiomedSciAI/Geno4SD/tree/main/geno4sd/ml_tools/rubricoe with tutorial at https://github.com/BiomedSciAI/Geno4SD/blob/main/tutorials/RubricOE.ipynb, and assistance through Filippo Utro (futro@us.ibm.com).
• Other: Any additional information required to reanalyze the data reported in this paper is available from the lead contact upon request.

### Method details

#### Detailed description of RubricOE workflow

We read plink files and denote the genotype data array by $X_{original}$ with sample IDs $LX_{original}$, and a comma-separated-values file with phenotype information $Y_{original}$, and sample IDs $LY_{original}\text{.}$ In this case, we will be dealing with a binary classification task, and so we call the labels case, specified by 1, and control, specified by 0.

We introduce some notations: For an ordered set *S* we will write S[i] to denote its *i*-th element, starting from zero, and for an ordered set of integers $I=\left\{i_{1},\ldots ,i_{n}\right\}$ we will write S[I] to denote the ordered set $\left\{S\left[i_{1}\right],\ldots ,S\left[i_{n}\right]\right\}$. The significance of this ordering is that it will allow for numeric iteration over relevant datasets.

We implicitly identify an ordered set of vectors $S=\left\{v_{1},\ldots ,n_{n}\right\}$, $v_{i}\in R^{N}$ with a matrix that has those vectors, in that order, as its rows. Thus S[i] also denotes the *i*-th row of the matrix *S*, and S[I] also denotes a matrix constructed by considering only rows with indices in *I*, keeping *I*’s ordering. Similarly, given a matrix *S* and ordered sets of integers I,J, S[I,J] denotes the matrix constructed by considering only rows with indices in *I* and columns in *J*, keeping the ordering in *I* and *J*.

Formally, the index functions are given by bijections

$$LX_{original}\overset{l_{X_{original}}}{\to }X_{original}$$

$$LY_{original}\overset{l_{Y_{original}}}{\to }Y_{original}$$

Note that rows in $X_{original}$ and $Y_{original}$ are not necessarily in the same order; furthermore: on plink files row contents and their associated sample IDs are kept in separate files, albeit with enough information to define $l_{X_{original}}$. We assume that if the ID information is thought of as a pair of sets, then

$$L_{complete}=LX_{original}\cap LY_{original}\neq \emptyset$$

and we impose a fixed ordering on $L_{complete}$, so that we can enumerate

$$L_{complete}=\left\{l_{0},l_{1},\ldots ,l_{n_{samples}-1}\right\}\text{.}$$

Here is a diagram of what we have so far:

$$\begin{array}{ccccc} L_{complete} & \to & LX_{original} & \underset{l_{X_{original}}}{\to } & X_{original}\\ & ↘ & LY_{original} & \underset{l_{Y_{original}}}{\to } & Y_{original} \end{array}$$

We now consider the sets

$$X_{complete}=l_{X_{original}}\left(L_{complete}\right)$$

$$Y_{complete}=l_{Y_{original}}\left(L_{complete}\right)\text{,}$$

thus taking only genotype and phenotype information for which sample IDs are available.

Since we have an ordering on $L_{complete}$, we can refer to the *first*, *second*, etc. sample, both in terms of genotype or phenotype information, by considering for genotype data the vectors $X_{complete}\left[i\right]$, and $Y_{complete}\left[i\right]$ for phenotype data.

For rubricOE, $X_{complete}$ and $Y_{complete}$ are the starting data (here distinguished from the originally unmatched phenotype-genotype data that potentially has more samples but no labels or viceversa). To further decouple possible identifying characteristics of the data, we *do not* use sample IDs beyond this point.

We then split $X_{complete}$ and $Y_{complete}$ into pairs $X_{working}$, $X_{validation}$ and $Y_{working}$, $Y_{validation}$, respectively, where *w* and *v* are short for *working* and *validation* data. Other than knowing the IDs of samples in the validation set, rubricOE does not have information on those samples, and will only use $X_{working},Y_{working}$ for all computations. Let us also denote by $n_{features}$ the number of columns of $X_{working}$, and we define $I_{working}=\left\{0,1,\ldots ,\left|X_{working}\right|\right\}$ as the set of indices and $I_{features}=\left\{0,\ldots ,n_{features}-1\right\}$ as the features (or columns) indices.

Assume that we have defined

(1) Ranking number of folds ds $n_{fol}$
(2) Ranking test size proportion <1$0<s_{test}$
(3) Scoring number of folds ds $m_{fol}$
(4) Scoring test size proportion <1$0<t_{test}$
(5) Number of curve steps $n_{steps}$,

And set

$$n_{increment}=\lfloor \frac{n_{features}}{n_{steps}}\rfloor \text{.}$$

We now describe rubricOE’s flow (see Figure S1C), with the following steps performed in each iteration.

(1) Generate $n_{folds}$ splits $\left(I_{train},I_{test}\right)$ of $I_{working}$. These splits are generated by randomly choosing $s_{test}\cdot \left|I_{working}\right|$ elements of $I_{working}$ to serve as indices of test elements and, for each fold, we define
(2) in $X_{train}=X_{working}\left[I_{tra}\right]$
(3) st $X_{test}=X_{working}\left[I_{te}\right]$
(4) in $Y_{train}=Y_{working}\left[I_{tra}\right]$
(5) st $Y_{test}=Y_{working}\left[I_{te}\right]$
(6) (note that we are omitting indices indicating current folds to reduce notation clutter). In addition we require that these sets have the same proportion of case/control samples in g $Y_{workin}$.
(7) For each fold, we fit a regressor (Ridge regression with error bars, in our particular case) to $\left(X_{train},Y_{train}\right)$. As a result we obtain for $0\leq i<n_{folds}$, $0\leq j<n_{feat}$ coefficients $c_{i,j}>0$ that represent the *importance* ascribed to each one of the features by the regressors.
(8) Define $c_{j}=\sum_{i}c_{i,j}/n_{folds}$. We assign importance in decreasing order to each feature by considering a stable permutation $\sigma :I_{features}\to I_{features}$ such that $\sigma \left(j_{1}\right)>\sigma \left(j_{2}\right)$ if and only if $c_{j_{1}}<c_{j_{2}}$. The interpretation of this is that feature *j* is the σ(j)-th feature ranked by importance in decreasing order, according to the regressor. Equivalently, feature $\sigma^{-1}\left(j\right)$ is the *j*-th feature ranked by importance in decreasing order.
(9) We consider a nested sequence of subsets of features $F_{0},\ldots ,F_{n_{steps}-1}$. For $0\leq i<n_{increment}-1$ these sets are defined by

$$F_{i}=\left\{\sigma^{-1}\left(0\right),\sigma^{-1}\left(1\right),\ldots ,\sigma^{-1}\left(\left(i+1\right)\cdot n_{increment}-1\right)\right\}$$

and we additionally define

$$F_{n_{steps}-1}=\left\{\sigma^{-1}\left(0\right),\sigma^{-1}\left(1\right),\ldots ,\sigma^{-1}\left(\left(i+1\right)\cdot n_{features}-1\right)\right\}\text{.}$$

(the reason behind the final special case is that sometimes the number of features is not divisible by the step size).

(1) As in Step 1, for each fold $r\in \left\{0,\ldots ,m_{folds}-1\right\}$ we generate balanced splits $\left(J_{r,train},J_{r,test}\right)$ of $I_{working}$. These splits are generated by randomly choosing $t_{test}\cdot \left|X_{working}\right|$ matching elements of $X_{working}$ and $Y_{working}$. For each fold $r\in \left\{0,\ldots ,m_{folds}-1\right\}$ and $i\in \left\{0,\ldots ,n_{steps}-1\right\}$, we define
(2) Fi $Z_{i,r,train}=X_{working}\left[J_{r,train}\text{,}\right]$
(3) Fi $Z_{i,r,test}=X_{working}\left[J_{r,test}\text{,}\right]$
(4) in $W_{r,train}=Y_{working}\left[J_{tra}\right]$
(5) st $W_{r,test}=Y_{working}\left[J_{te}\right]$
(6) For each $i\in \left\{0,\ldots ,n_{steps}-1\right\}$, we fit a classifier *C* (here we use an SVM with linear kernel) to $\left(Z_{i,r,train},W_{train}\right)$. We record *C*’s performance on $\left(Z_{i,r,test},W_{test}\right)$ in terms of its Youden index, denoted by r $y_{i,}$.
(7) For $i\in \left\{0,\ldots ,n_{steps}-1\right\}$ define

$$y_{i}=\frac{\sum_{r=0}^{m_{folds}}y_{i,r}}{m_{folds}}$$

Thus, by the end of the execution of these steps, we get an ordered set $\left\{y_{0},\ldots ,y_{n_{steps}-1}\right\}$ which we call the *Youden curve* $K_{i}$ of the *i*-th iteration. We now compute, for all Youden curves $K_{i}$, the first (from left to right) local maximum of $K_{i}$, here a “local maximum” *x* at index $j_{\max}$ of $K_{i}$ is understood as an element such that $K_{i}\left[j_{\max}-1\right]\leq x\leq K_{i}\left[j_{\max}+1\right]$, and we define. The output of RubricOE are the counts of each feature appearing in each of the iterations’ curve before its corresponding local maximum.

#### Data processing and downstream analysis

Data were acquired on June 26, 2021 from the Alzheimer’s Disease Sequencing Project (ADSP) repository at National Institude of Aging Alzheimer’s Data Storage Site (https://www.niagads.org/adsp/), comprised of data not excluded for use by commercial entities,^17^ release R3 from 14474 subjects consenting to access by commercial entities, excluding family set data, retaining 78815437 SNPs marked as “PASS” by variant quality score recalibration in the GATK suite. SNPs showing systematic missingness were identified and filtered. Phenotypes available in the dataset include ethnicity, race, age, sex, AD status, whether diagnosed on enrollement, acquired it during the study, BRAAK stage, the APOE4 genotype (determined separately from the WGS analysis). We retained age, sex, and AD status. Females comprised 63.3%, 6,446 of the samples, with 45.9%, AD 4365 diagnosed subjects. The average age of the subjects was 75.4 years, with a standard deviation of 8.9 years.

For the dataset, MAF was filtered at 0.05, LD filtered with window size of 50 kb, step size 5, $r^{2}$ threshold of 0.5, and HWE threshold of $1\times 10^{-12}$. Beagle was applied for imputation.^18^^,^^19^

LDpred2 includes a QC protocol involving LD, etc. The same standard was applied to the data processed by RubricOE.

Analysis applying RubricOE (here) and LDpred2^11^ are described in the introduction sections describing RubricOE and the application of LDpred2. Quantitative estimation of the predictive power of feature sets selected by RubricOE and LDpred2 for comparative purposes were evaluated using Sklearn’s^16^ sklearn.svm.SVC with default settings in order to contrast AUC and $F_{1}$ statistics for leading features identified and ranked by RubricOE and LDpred2. p-values for feature predictions were estimated using pytyon statsmodels logistic regression.

### Quantification and statistical analaysis

Autosomal SNP alleles are diploid with values “A,” “C,” “G,” and “T.” Any record with a haploid SNP was excluded. Other QC steps were outlined in STAR methods. RubricOE assigns ranking and selection to predictive sets based on how many cross-validation realizations the SNP appears in the set of discriminating SNPs. LDpred2 assigns weights β to SNPs, which may be used to rank the contributions of each SNP to its predictivity model. Standard GWAS logistic regression was applied to each SNP using python statsmodels yielding odds ratios and p values.

RubricOE and LDpred were applied to predict binary valued phenotypes indicating whether a subject did or did not have Alzheimer’s disease. For a control, the phenotypes were shuffled to show how random, non-genetic diseases appear under these analyses.

The lists of leading SNPs identified by RubricOE and LDpred2 were selected according to a support threshold of 40 of 50 for RubricOE and the top 50% of the range of β scores for LDpred2. The largest LDpred2 β reflects how well the SNPs predict the phenotype.

“Models” are defined by the representative SNPs identified by RubricOE and LDpred2. The predictive power of these SNPs was estimated using a linear kernel support vector machine from the python sklearn package. The results were represented by AUC and $F_{1}$ scores.

Therefore,

(1) **Phenotypes** are binary valued data items indicating if a subject has or does not have Alzheimer’s disease. A control set was generated by shuffling the phenotype-to-subject assignments. Shuffling was not used as a way to estimate variations of the null hypothesis, but was used to characterize a null model.
(2) **SNPs** are discrete valued diploid entities characterized by logistic-regression p values, RubricOE support (integers up to 50 cross-validation replications), and LDpred2 β scores, and a binary value of whether the SNP was selected by LDpred2 or RubricOE according to threshold.
(3) **Models** are lists of SNPs selected by RubricOE or LDPred2. They are characterized by AUC and L1 measures of predictivity reported by a linear kernel support vector machine in python’s sklearn library.

## Supplemental information

Document S1. Figure S1 and Tables S2–S5

Table S1. SNPS with support ≥ 40 from Chr 11, related to Figure 4
