# Supplementary material for: AI-enabled evaluation of genome-wide association relevance and polygenic risk score prediction in Alzheimer's disease
Source: iScience. 2024 Feb 12;27(3):109209. doi: 10.1016/j.isci.2024.109209 (PMC10910245; doi:10.1016/j.isci.2024.109209)
Supplement: Document S1. Figure S1 and Tables S2–S5 [file mmc1.pdf]

iScience, Volume *27*

Supplemental information

AI-enabled evaluation of genome-wide association relevance and polygenic risk score prediction in Alzheimer's disease

Daniel E. Platt, Aldo Guzmán-Sáenz, Aritra Bose, Subrata Saha, Filippo Utro, and Laxmi Parida

Supplementary Figures

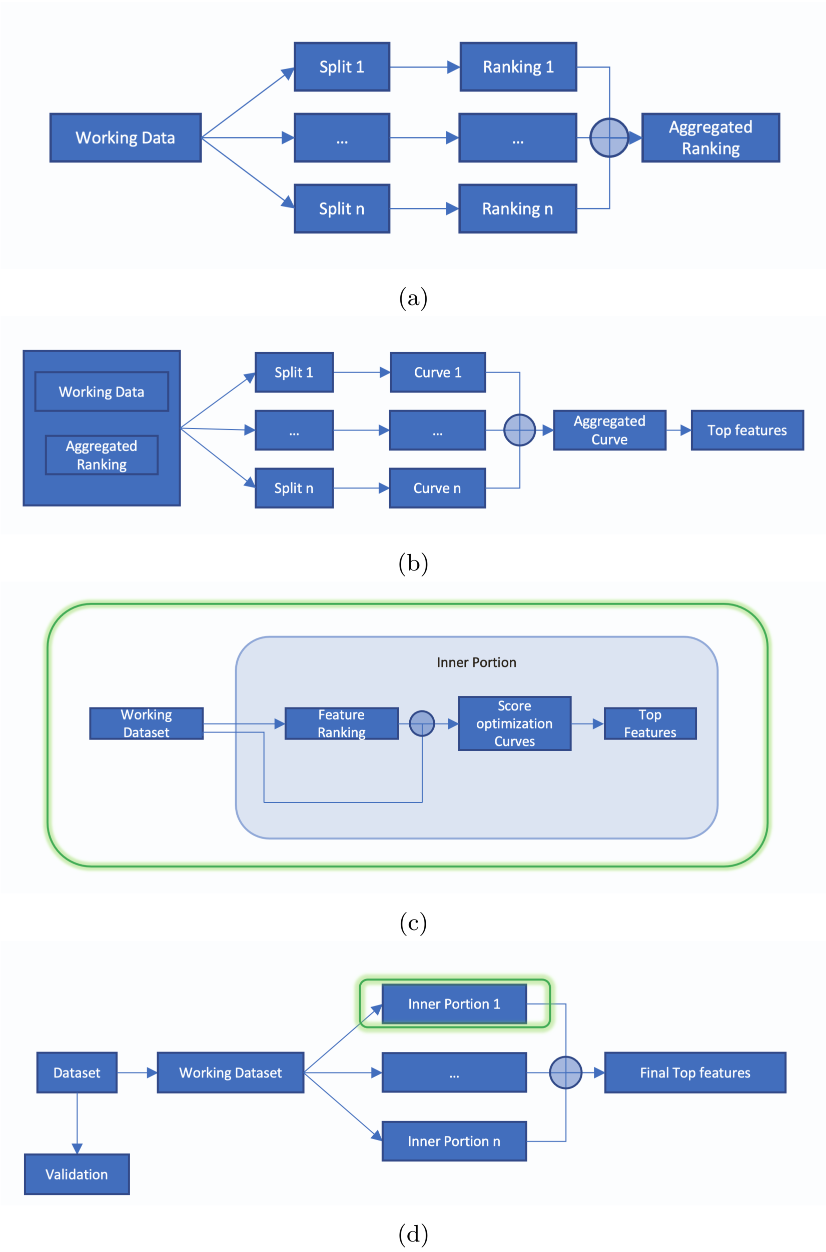

Figure S1: Flowcharts describing feature ranking, selection, scoring, and cross-validation replications. (a) RubricOE ranking step's execution flow, (b) RubricOE scoring step's execution flow, (c) RubricOE main loop's body, (d) Overview of RubricOE complete execution flow. Related to Figure 1.

Supplementary Tables

Table S2: SNPS with support ≥ 40 from Chr 19. Related to Figure 5.

SNP	Gene	OR	95%CI-	95%CI+	P-val	Support
rs1160985		1.4690	1.3870	1.5558	2.194e-39	50
rs10119	APOE	0.6021	0.5659	0.6406	6.14e-58	50
rs741780		1.4677	1.3858	1.5543	3.029e-39	50
rs1038026		1.4661	1.3844	1.5526	4.048e-39	50
rs1038025		1.4680	1.3861	1.5549	3.391e-39	50
rs12721051	APOC1	0.4935	0.4589	0.5307	8.892e-81	50
rs438811	APOC1	0.6734	0.6331	0.7162	2.885e-36	50
rs62115563	KDM4B	0.8286	0.7633	0.8995	7.094e-06	50
rs769449	APOE	0.4822	0.4425	0.5254	2.424e-62	50
rs157582		0.7284	0.6848	0.7747	7.125e-24	50
rs184017		0.7303	0.6864	0.7769	2.706e-23	50
rs56131196	APOC1	0.5520	0.5151	0.5916	1.951e-63	50
rs1081105	APOE	0.4915	0.4276	0.5650	1.756e-23	50
rs4420638	APOC1	0.5569	0.5197	0.5967	6.666e-62	50
rs4452075	ZNF527	1.1926	1.1204	1.2694	3.263e-08	50
rs115881343		0.4975	0.4316	0.5734	5.674e-22	50
rs112849259		0.4900	0.4236	0.5668	7.458e-22	50
rs483082	APOC1	0.6855	0.6440	0.7298	2.499e-32	50
rs79398853		0.4913	0.4245	0.5686	1.546e-21	50
rs7259620	APOE	1.3864	1.3075	1.4701	8.604e-28	50
rs114536010		0.4924	0.4254	0.5699	2.108e-21	50
rs2459634		1.3058	1.2297	1.3866	3.008e-18	50
rs484195	APOC1	1.2020	1.1308	1.2776	3.425e-09	50
rs141846480		0.7475	0.6380	0.8758	0.0003168	49
rs6508719	ZNF527	1.1956	1.1231	1.2728	2.165e-08	49
rs7250946		0.7435	0.6311	0.8761	0.0003995	49
rs116881820		0.5017	0.4340	0.5800	1.13e-20	49
rs77301115		0.5031	0.4353	0.5815	1.443e-20	49
rs10414043	APOC1	0.6226	0.5773	0.6714	8.431e-35	49
rs157595		1.2008	1.1297	1.2764	4.193e-09	49
rs405697		1.1829	1.1038	1.2677	1.978e-06	49
rs1559229	ZNF527	1.1962	1.1236	1.2734	1.991e-08	48
rs73041616		0.9050	0.7745	1.0575	0.2091	48
rs11084395		0.9024	0.8514	0.9564	0.0005394	48
rs4804749		0.8322	0.7799	0.8881	2.996e-08	47
rs111789331		0.5351	0.4942	0.5794	1.533e-53	47
rs79539512		0.7536	0.6393	0.8883	0.0007478	47
rs73015715	FCHO1	1.0165	0.9545	1.0825	0.6111	47
rs73015714	FCHO1	1.0206	0.9584	1.0868	0.5254	47
rs117191274		0.7511	0.6372	0.8852	0.0006407	46
rs55714539		0.9133	0.8596	0.9704	0.003364	46
rs4803460		1.1967	1.1242	1.2738	1.769e-08	45
rs12104396		0.9422	0.8904	0.9970	0.03906	45
rs2190799		1.0902	1.0301	1.1537	0.002819	45
rs2238628	GNA15-DT	0.9870	0.8885	1.0966	0.8082	45

rs59007384		0.7231	0.6772	0.7721	3.49e-22	45
rs112692857		0.8228	0.7619	0.8887	6.816e-07	43
rs2335089		1.0396	0.9734	1.1103	0.2477	43
rs11878905	LOC390877	0.9030	0.8530	0.9559	0.0004416	43
rs56208000	FAAP24	1.1006	1.0310	1.1749	0.004043	43
rs3760800	SULT2B1	0.8972	0.8479	0.9493	0.0001647	42
rs184926367		0.7566	0.6421	0.8915	0.0008607	42
rs79846541	CREB3L3	1.5313	1.1009	2.1299	0.01138	42
rs59181603		1.0041	0.9383	1.0745	0.9056	42
rs4807413	GNA15-DT	1.0498	0.9422	1.1697	0.3787	42
rs12979832	ZNF552	0.9705	0.9127	1.0319	0.3387	42
rs59559975	PLIN3	0.8424	0.7605	0.9331	0.001014	42
rs190415956	PIK3R2	1.3243	1.0224	1.7154	0.03338	41
rs3212701	JAK3	1.0796	1.0176	1.1453	0.01108	41
rs36108309	FCER2	0.9000	0.7945	1.0194	0.09736	41
rs80332649		0.7559	0.6410	0.8914	0.0008771	41
rs17714931	MIR4531	0.7559	0.6410	0.8914	0.0008771	41
rs369736		0.9539	0.8935	1.0183	0.157	41
rs73050216	NECTIN2	1.1295	1.0400	1.2268	0.003864	41
rs73013530		1.1513	0.9940	1.3335	0.06023	41
rs17760970		1.0768	1.0166	1.1406	0.01171	41
rs11668327		1.0447	0.9528	1.1455	0.3517	41
rs11668338	FCER2	0.8942	0.7896	1.0126	0.07793	40
rs11672507	FCER2	0.9049	0.8002	1.0233	0.1113	40
rs140172136		0.6286	0.5135	0.7696	6.926e-06	40
rs117647142		0.6286	0.5135	0.7696	6.926e-06	40
rs67965336		0.8260	0.7648	0.8921	1.134e-06	40
rs2073614	SNORD35B	1.1716	1.0932	1.2557	7.506e-06	40
chr19:54301977		0.8292	0.7774	0.8844	1.265e-08	40
rs439401		1.1932	1.1223	1.2686	1.566e-08	40
chr19:54303249		0.8280	0.7762	0.8832	9.864e-09	40

Table S3: Shuffled phenotypes, SNPS with support ≥ 40 from Chr 19. Related to Figure 5.

SNP	Gene	OR	95%CI-	95%CI+	P-val	Support
rs2682590		1.2294	1.1486	1.3159	2.65e-09	50
rs7258524		0.9883	0.9164	1.0659	0.7604	50
rs112791293		1.2689	1.1675	1.3790	2.055e-08	50
rs766655		1.0324	0.9754	1.0927	0.2717	50
rs62103074		1.1322	1.0704	1.1976	1.465e-05	49
rs4805064		1.1250	1.0633	1.1903	4.307e-05	49
rs4805961		1.1250	1.0633	1.1903	4.307e-05	49
rs7246868		1.1253	1.0636	1.1907	4.11e-05	49
rs976032		1.1254	1.0636	1.1907	4.079e-05	49
rs976031		1.1244	1.0627	1.1896	4.667e-05	49
rs4805958		1.1324	1.0706	1.1979	1.425e-05	49
rs59604612		1.2971	1.1686	1.4398	1.023e-06	48

rs62119263		0.8826	0.8158	0.9549	0.001875	48
rs12971352		1.0825	1.0099	1.1603	0.02531	47
rs11670672		0.9555	0.8938	1.0215	0.182	47
rs382592		0.9740	0.9200	1.0312	0.366	47
rs1905475		1.0283	0.9714	1.0884	0.3365	47
rs4806416		0.8885	0.8402	0.9396	3.391e-05	47
rs2195965		0.9960	0.9407	1.0546	0.8912	46
rs12608491		0.9968	0.9414	1.0554	0.9124	46
rs12151204	ZNF431	1.0504	0.9647	1.1437	0.2573	46
rs3730256		1.0617	0.9646	1.1686	0.2214	46
rs7258975	ZNF160	0.9990	0.9408	1.0608	0.9733	46
rs7258976	ZNF160	1.0002	0.9419	1.0622	0.9943	46
rs62103068		0.9544	0.8927	1.0203	0.1707	45
rs10405676	LOC105372256	1.0672	1.0025	1.1361	0.0415	45
rs2082479		0.9976	0.9423	1.0563	0.9355	45
rs148313465		0.7664	0.6108	0.9617	0.0216	44
rs2082480		0.9976	0.9423	1.0563	0.9355	44
rs4932986		0.9972	0.9418	1.0558	0.923	44
rs58170323		0.9980	0.9426	1.0567	0.9462	44
rs57501688		0.9980	0.9426	1.0567	0.9462	44
rs4932759		0.9972	0.9418	1.0558	0.923	44
rs12976546	LINC02841	0.9387	0.8822	0.9988	0.0457	44
rs62103289	LINC02841	0.9426	0.8859	1.0030	0.06195	44
rs7259215		1.0682	0.9956	1.1461	0.06637	43
rs2195964		1.0018	0.9461	1.0608	0.9511	43
rs2099354		0.9967	0.9414	1.0553	0.9105	43
rs62128036	SIN3B	0.9160	0.8429	0.9955	0.03885	42
rs12972423	LINC02841	0.9423	0.8856	1.0027	0.06062	42
rs11881107	ZNF431	1.0793	0.9978	1.1673	0.05671	42
rs62118220		0.8709	0.8066	0.9404	0.0004184	42
rs8110234	KDM4B	1.1434	1.0657	1.2268	0.0001896	42
rs13345062	MVB12A	1.0624	0.9879	1.1425	0.1029	41
rs2313234	SIN3B	0.9159	0.8429	0.9951	0.03797	41
rs4932760		1.0023	0.9465	1.0612	0.9385	41
rs1230295		0.9045	0.8541	0.9578	0.0005914	41
rs12462195		0.8852	0.8373	0.9359	1.795e-05	41
rs11084754		1.1452	1.0830	1.2109	1.931e-06	41
rs8182484		0.9577	0.8876	1.0333	0.2645	41
rs73049659		0.8752	0.8077	0.9484	0.001139	41
rs10405052	KDM4B	1.1406	1.0635	1.2233	0.00023	41
rs1368444		1.0616	0.9102	1.2382	0.4461	41
rs181380437		0.5876	0.3522	0.9804	0.04177	40
rs17836364	LILRA4	0.9491	0.8798	1.0238	0.1763	40
rs2682589		1.2706	1.1815	1.3665	1.112e-10	40
rs4804476	OLFM2	1.1611	1.0929	1.2336	1.333e-06	40
rs428729		0.9587	0.9055	1.0151	0.1479	40
rs73032697		0.9582	0.8882	1.0339	0.2711	40
rs2291142	KDM4B	1.1426	1.0651	1.2257	0.0001993	40

Table S4: RubricOE on PRS QC, SNPS with support ≥ 40 from Chr 19. Related to Figure 5.

SNP	Gene	OR	95%CI-	95%CI+	P-val	Support
rs438811	APOC1	0.6733	0.6330	0.7161	2.705e-36	50
rs4420638	APOC1	0.5570	0.5198	0.5969	7.481e-62	50
rs10414043	APOC1	0.6227	0.5775	0.6716	9.086e-35	50
rs283815	NECTIN2	0.7248	0.6820	0.7704	4.679e-25	50
rs11668327		1.0450	0.9530	1.1458	0.3494	50
rs10119	APOE	0.6022	0.5661	0.6407	7.029e-58	50
rs116286867		0.9215	0.7290	1.1649	0.4943	50
rs7412	APOE	1.6730	1.4494	1.9312	2.08e-12	50
rs76887317	LILRB3	1.0437	0.9691	1.1241	0.2583	50
rs434132	APOE	0.7791	0.6766	0.8970	0.0005159	50
rs157591		0.8125	0.7011	0.9416	0.005782	50
rs1038026		1.4656	1.3840	1.5521	4.755e-39	50
rs190712692		1.5981	1.3602	1.8775	1.188e-08	50
rs143613107		1.6156	1.2725	2.0513	8.216e-05	50
rs8110296	tmem62	1.2982	1.1487	1.4672	2.917e-05	50
rs75673554	PTPRS	1.2538	1.1153	1.4096	0.0001523	50
rs1126454		0.8792	0.8303	0.9310	1.052e-05	50
rs7250112	CD209	1.4368	1.2750	1.6192	2.782e-09	49
rs202126014	RAB8A	2.4163	1.7661	3.3057	3.452e-08	49
rs113949386		1.6981	1.3045	2.2106	8.312e-05	49
rs7252828		0.9760	0.9221	1.0331	0.4021	49
rs78566095		0.6862	0.6198	0.7598	4.405e-13	49
rs142533877		1.3981	1.0590	1.8459	0.01806	49
rs183552722	ZNF765	1.3834	1.0624	1.8014	0.01599	48
rs145184198	LOC116276494	0.7588	0.6867	0.8385	6.083e-08	48
rs11881698		1.0814	1.0222	1.1441	0.006456	48
rs75178253		1.0325	0.9119	1.1689	0.6138	48
rs73022604		1.2729	1.1279	1.4365	9.237e-05	48
rs373304918		2.5649	1.8791	3.5011	2.966e-09	48
rs188216716		0.9064	0.7172	1.1455	0.4106	48
rs150092223		0.7680	0.6082	0.9698	0.02658	48
rs11666071		0.8252	0.7797	0.8735	3.392e-11	48
rs157583		0.8283	0.7201	0.9526	0.008297	47
rs73615268		1.8183	1.4556	2.2714	1.382e-07	47
rs184839528	CGB5	0.8976	0.7153	1.1263	0.3509	47
rs10425116		1.5478	1.3631	1.7576	1.633e-11	47
rs112803379		1.5139	1.1546	1.9849	0.002697	47
rs75501048	JAK3	2.1308	1.6936	2.6808	1.072e-10	47
rs117031587	LOC107985320	0.6174	0.4902	0.7775	4.165e-05	46
rs8109131	INSR	0.9025	0.8521	0.9557	0.0004536	46
rs1671200	GP6-AS1	0.8251	0.7741	0.8795	3.57e-09	46
rs148707101		0.5480	0.4291	0.6999	1.441e-06	46
rs73046490		0.7679	0.6287	0.9380	0.009668	46
rs10413822	CACNA1A	1.9486	1.5680	2.4217	1.778e-09	46

rs147228142	ACER1	1.0379	0.8269	1.3027	0.7485	45
rs78410670	CEACAM22P	0.6644	0.5745	0.7683	3.488e-08	45
rs184926367		0.7567	0.6422	0.8916	0.000868	45
rs112440167	ZNF585A	1.0098	0.8310	1.2271	0.9217	45
rs2384685		1.1330	1.0694	1.2004	2.288e-05	45
rs729078		1.1213	0.9975	1.2604	0.05511	45
rs148276553		1.1064	0.9735	1.2574	0.1214	44
rs151179818	MBOAT7	1.6039	1.2202	2.1083	0.0007086	44
rs55960616	ZNF558	0.6996	0.6073	0.8059	7.455e-07	44
rs4806664	TMEM150B	1.2388	1.1682	1.3137	8.43e-13	43
rs16981094		1.5635	1.3144	1.8599	4.492e-07	43
rs17714931	MIR4531	0.7561	0.6412	0.8916	0.0008845	43
rs11880658		0.9542	0.8107	1.1230	0.5725	43
rs111339234	ZNF98	1.0985	0.9020	1.3379	0.3502	43
rs73934829		1.0675	0.8595	1.3258	0.5549	43
rs142267272	NLRP12	0.5172	0.3895	0.6867	5.179e-06	43
rs144104132		0.6197	0.4881	0.7869	8.619e-05	42
rs73928313		1.6784	1.3402	2.1019	6.456e-06	42
rs117125065	SLC6A16	0.6649	0.5548	0.7969	1.004e-05	42
rs117746398	ZNF283	0.7163	0.5824	0.8810	0.001576	42
rs116590874		1.4214	1.0956	1.8441	0.008118	42
rs112473731		1.7348	1.2898	2.3334	0.0002702	42
rs142299057		0.5513	0.4471	0.6797	2.51e-08	42
rs79890159		0.7308	0.6562	0.8138	1.127e-08	42
rs76654869		1.7333	1.3634	2.2035	7.099e-06	42
rs112716951	cpped1	0.8140	0.7571	0.8751	2.565e-08	41
rs73926011	ZNF626	1.5851	1.3533	1.8566	1.13e-08	41
rs10410968		0.9747	0.7828	1.2136	0.8188	41
rs166907	NECTIN2	0.8820	0.7582	1.0260	0.1038	41
rs8110017	DMWD	1.0332	0.9535	1.1196	0.4249	41
rs187595051		0.7838	0.6681	0.9195	0.00279	41
rs146681582	NLRP12	1.0678	0.9876	1.1544	0.09959	41
rs112584199	NFKBID	1.7009	1.2882	2.2458	0.0001798	41
rs141846480		0.7476	0.6381	0.8760	0.0003198	41
rs76022393		1.7004	1.3623	2.1224	2.691e-06	41
rs8106692		0.9466	0.8943	1.0020	0.05852	41
rs2446368		1.2283	1.1102	1.3590	6.671e-05	40
rs12462913		1.0610	0.9580	1.1752	0.2559	40
rs28446219		1.0288	0.9506	1.1134	0.4816	40
rs2041194		0.8202	0.7685	0.8753	2.308e-09	40
rs112113930		0.8550	0.7753	0.9428	0.001691	40
rs2109043		0.9674	0.7582	1.2344	0.7899	40
rs146021196		0.9674	0.7582	1.2344	0.7899	40
rs140480140	APOC1	0.5213	0.4546	0.5977	1.033e-20	40
rs73015326		1.1873	1.0102	1.3954	0.03728	40
rs12462664	RFPL4AL1	0.9599	0.9059	1.0171	0.1659	40
rs117808029		0.7068	0.5836	0.8562	0.0003883	40
rs185646906		2.0154	1.5329	2.6496	5.172e-07	40
rs2656880		1.1366	0.9727	1.3281	0.107	40

rs111442269	SMIM7	0.8645	0.7123	1.0493	0.1407	40

Table S5: LDPred2 on PRS QC from Chr 19. Related to Figure 5.

SNP	Gene	OR	95%CI-	95%CI+	P-val	β
rs438811	APOC1	0.6733	0.6330	0.7161	2.705e-36	0.007505
rs34482266	MIR521-2	1.4776	1.3051	1.6730	7.097e-10	0.010272
rs74431057	LOC107985303	0.5880	0.4609	0.7503	1.956e-05	0.013185
rs139175392		1.7508	1.3315	2.3020	6.066e-05	0.010482
rs140172136		0.6288	0.5136	0.7698	6.988e-06	0.007633
rs117299550		0.6581	0.5498	0.7876	5.006e-06	0.007263
rs117409064		1.2609	1.0592	1.5009	0.009146	0.007043
rs117015683	FCHO1	0.5780	0.4607	0.7251	2.157e-06	0.010356
rs74482662	CACNA1A	1.5258	1.3319	1.7478	1.098e-09	0.01118
rs80062066	VAV1	0.6531	0.5350	0.7972	2.812e-05	0.00697
rs12462579		1.5565	1.4044	1.7250	3.331e-17	0.01063
rs117606744	THEG	0.6048	0.4661	0.7849	0.0001563	0.012866
rs58881629		1.2901	1.1793	1.4113	2.727e-08	0.007244
rs4420638	APOC1	0.5570	0.5198	0.5969	7.481e-62	0.010071
rs146392411		0.7397	0.6646	0.8232	3.3e-08	0.008538
rs718385		1.8117	1.5406	2.1304	6.629e-13	0.010527
rs62130538	SLC8A2	1.3019	1.0950	1.5477	0.002803	0.008669
rs10119	APOE	0.6022	0.5661	0.6407	7.029e-58	0.012024
rs139166449	ZNF56P	1.3676	1.0804	1.7311	0.00924	0.009287
rs78410670	CEACAM22P	0.6644	0.5745	0.7683	3.488e-08	0.009173
rs187737536		0.8101	0.6835	0.9601	0.01512	0.007438
rs150896709		0.6745	0.5259	0.8650	0.001921	0.007494
rs141323496	PLEKHA4	0.6179	0.4898	0.7795	4.889e-05	0.010581
rs142267272	NLRP12	0.5172	0.3895	0.6867	5.179e-06	0.010761
rs78809666		1.2502	1.0113	1.5457	0.03905	0.00668
rs10415225		1.0477	0.9111	1.2046	0.5135	0.006971
rs148707101		0.5480	0.4291	0.6999	1.441e-06	0.016995
rs4802772	CD33	1.2484	1.1350	1.3731	4.922e-06	0.008603
rs116945692		1.2973	1.0980	1.5328	0.002224	0.007553
rs79792478		1.7240	1.4315	2.0762	9.356e-09	0.007459
rs12971815		1.2345	1.0737	1.4194	0.003091	0.007763
rs4897855		1.4681	1.2484	1.7264	3.434e-06	0.007083
rs116863072		0.6046	0.4704	0.7772	8.561e-05	0.007023
